# Alginate Beads with Encapsulated Bioactive Substances from *Mangifera indica* Peels as Promising Peroral Delivery Systems

**DOI:** 10.3390/foods13152404

**Published:** 2024-07-29

**Authors:** Nika Kučuk, Mateja Primožič, Željko Knez, Maja Leitgeb

**Affiliations:** 1 Faculty of Chemistry and Chemical Engineering, University of Maribor, Smetanova Ulica 17, 2000 Maribor, Slovenia; nika.kucuk@um.si (N.K.); mateja.primozic@um.si (M.P.); zeljko.knez@um.si (Ž.K.); 2 Faculty of Medicine, University of Maribor, Taborska Ulica 8, 2000 Maribor, Slovenia

**Keywords:** alginate beads, encapsulation, *Mangifera indica*, mango peels, characterization, in vitro release, antibacterial activity

## Abstract

Since various bioactive substances are unstable and can degrade in the gastrointestinal tract, their stabilization is crucial. This study aimed to encapsulate mango peel extract (MPE) into edible alginate beads using the ionotropic gelation method for the potential oral delivery of bioactive substances. Mango peels, generally discarded and environmentally harmful, are rich in health-promoting bioactive substances. The alginate beads were examined for entrapment efficiency, particle size, morphology, thermal stability, physiochemical interactions, release profile under gastrointestinal conditions, and antibacterial efficacy. The study demonstrated the successful encapsulation of MPE with an efficiency of 63.1%. The in vitro release study showed the stability of the alginate beads in simulated gastric fluid with a maximum release of 45.0%, and sustained, almost complete release (99.4%) in simulated intestinal fluid, indicating successful absorption into the human body. In both fluids, the MPE release followed first-order kinetics. Encapsulation successfully maintained the antibacterial properties of MPE, with significant inhibitory activity against pathogenic intestinal bacteria. This is the first study on MPE encapsulation in alginate beads, presenting a promising oral delivery system for high-added-value applications in the food industry for dietary supplements, functional foods, or food additives. Their production is sustainable and economical, utilizing waste material and reducing environmental pollution.

## 1. Introduction

The demand for functional foods containing natural, biologically active compounds with health-promoting properties is constantly increasing. However, various bioactive substances can be broken down too quickly in the gastrointestinal tract. Therefore, their bioaccessibility and bioactivity are often limited. In order to incorporate sensitive natural bioactive substances into functional foods, their stability, viability, and biological activity must be maintained [1,2,3], which can be successfully ensured by encapsulation into various carriers. The encapsulation of bioactive substances is crucial for protection against pH, moisture, heat, and other harsh environmental conditions [4]. Therefore, encapsulated substances can reach the target sites, prevent possible toxic effects on healthy cells, and provide sustained and prolonged release [5]. Encapsulating substances in higher concentrations is also possible, enabling a better therapeutic effect through prolonged release. In this way, the encapsulated active ingredients are successfully protected from metabolic processes and thus retain their biological activity [6,7].

At the same time, there is a strong trend toward the use of biocompatible, biodegradable, and non-toxic delivery systems. Polymer-based particles are one of the most important carriers for various therapeutic agents as they are biodegradable, biocompatible, non-immunogenic, and non-toxic. A well-researched option is encapsulation in biopolymeric particles/microbeads such as alginate beads, which are particularly suitable for the food industry [8]. Alginate is a natural polysaccharide mainly derived from brown macroalgae [9] but can also be produced by some bacterial species such as *Azotobacter* spp. and *Pseudomonas* spp. [10]. Alginate consists of repeating blocks of β-D-mannuronic acid (M-blocks) and α-L-guluronic acid (G-blocks) linked by 1,4-glycosidic bonds [11]. In addition, alginate is also approved by the Food and Drugs Administration (FDA) as Generally Recognized as Safe (GRAS) for various applications, including food, pharmaceuticals, and medicine [12].

Alginate beads are formed by the ionotropic gelation method in the presence of divalent ions, in particular calcium (Ca^2+^) and magnesium ions (Mg^2+^), through a cross-linking mechanism. Cross-linking occurs through interactions between the G-blocks of the alginate polymer chains and divalent ions, which leads to the formation of a structure known as the “egg box” model [13,14]. The ionotropic gelation method is the most commonly used method for the production of stable polysaccharide particles [15,16]. This technique is also cost-effective, requiring only simple equipment, low operating temperature, and no organic solvents [17]. The produced alginate beads are usually large (more than 1 mm), which is due to the accumulation of the droplet volume at the nozzle tip. Nevertheless, the beads are generally very uniform in size [14]. 

Nowadays, agro-industrial waste from the fruit industry strongly contributes to a growing environmental problem through improper disposal and further non-use [18]. Mango (*Mangifera indica* L.) is one of the most important and popular tropical fruits, with excellent taste, aroma, and high phytonutrient content. As a result, 15 to 25 million tons of mango peels are generated every year. The disposal of these mango by-products in landfills leads to considerable environmental problems [19]. Due to their high biodegradability and fermentability, mango fruit peels can contribute to environmental issues such as water and soil pollution, eutrophication, global warming, and the greenhouse effect [20,21,22]. They also have a direct impact on living organisms in terms of climate change through the emission of greenhouse gasses and air pollution, which can affect respiratory health. The production of leachate and the process of eutrophication can damage aquatic ecosystems. Since mango peels are considered organic waste, they can also be a breeding ground for harmful pathogens that can transmit diseases to living organisms if not appropriately treated [23,24,25]. On the other hand, mango by-products, especially peels, represent an important source of bioactive compounds, which could be successfully isolated and further used for various health-benefit purposes in the food, cosmetic, and pharmaceutical industries. In addition, their use in different applications could reduce the amount of waste and the negative impacts on the environment and human health [24,26,27,28].

Natural extract–loaded alginate beads are promising for the production of functional foods as they are stable in a very acidic environment and degrade in an alkaline environment, leading to the successful release of the encapsulated bioactive substances and, thus, absorption in the intestine and utilization by the body [29,30]. Alginate beads produced by ionotropic gelation have been extensively studied in recent years for the encapsulation and, therefore, the stabilization of various natural extracts and bioactive substances. Encapsulation has proven to be a successful strategy to protect and control the release of various polyphenols (e.g., apigenin [31], curcumin [32], ellagic acid [33], gallic acid [34], and quercetin [35]) from alginate beads while improving their biological activity. Alginate beads also show improved functionality and stability of the encapsulated bioactive compounds of extracts from *Mesona chinensis* [36], *Spirulina* sp. LEB-18 [37], *Moringa oleifera* [38], and *Thymus serpyllum* [39]. The encapsulation of polyphenols, essential oils, and vitamins in carriers such as alginate beads is a successful strategy for preserving their antioxidant and antimicrobial activities [40]. Alginate beads with encapsulated extract of jabuticaba peels and propolis have also been used as alternative natural colorants for food and beverages due to their high content of anthocyanins—natural pigments with antioxidant properties [41]. The literature researched also revealed the use of alginate beads as carriers for the stability of extracts from various fruit waste parts, such as pomegranate peels [42], longan seed [43], annatto seed [44], lime peels [45], plum peels [46], and grape pomace [1]. Loading alginate beads with natural extracts significantly benefits human health by improving the extracts’ stability and shelf-life and allowing their controlled and sustained release at the target site. This results in increased bioavailability and improved bioactivity, contributing to better overall health outcomes [47]. However, mango peel extract (MPE) encapsulation in alginate beads has not yet been studied and published. Therefore, this is the first such study to be conducted.

Overall, the aim of our study was the production of edible alginate beads encapsulated with MPE to protect valuable bioactive substances from rapid degradation as a potential biodegradable carrier system for the production of high-value-added products with health-promoting properties, especially for the food industry. In addition, the physical properties of MPE-loaded alginate beads, entrapment efficiency, particle size, morphology, thermal stability, release profile under gastrointestinal conditions, and antibacterial efficacy against the Gram-negative bacterium *Escherichia coli* and the Gram-positive bacterium *Staphylococcus aureus* were investigated, which, to the best of our knowledge, has not been studied before.

## 2. Materials and Methods

### 2.1. Chemicals and Reagents

Ethanol (EtOH, ≥99.5%), hydrochloric acid (HCl, 37.0%), meat extract, meat peptone, potassium dihydrogen phosphate (KH_2_PO_4_), potassium iodide (KI), sodium chloride (NaCl), sodium dihydrogen phosphate monohydrate (NaH_2_PO_4_·H_2_O), and sodium hydrogen phosphate (Na_2_HPO_4_) were purchased from Merck, Darmstadt, Germany. Tryptic soy broth was obtained from Fluka, Buchs, Switzerland. Calcium chloride (CaCl_2_) and D-(+)-glucose anhydrous were purchased from Kemika, Zagreb, Croatia. Agar, yeast extract, peptone from soybean, sodium alginate, sodium carbonate (Na₂CO₃, ≥99.5%), and sodium hydroxide (NaOH, ≥95.0%) were purchased from Sigma-Aldrich, St. Louis, MO, USA. Ciprofloxacin (CIP, 400 mg/200 mL) was obtained from the University Medical Centre Maribor, Maribor, Slovenia.

### 2.2. Microorganisms

Selected bacterial species *E. coli* (DSM 498) and *S. aureus* (DSM 346) were purchased from DSMZ-German Collection of Microorganisms and Cell Cultures GmbH from Berlin, Germany.

### 2.3. Preparation and Extraction of Mango Peels

Ripe Keitt-type mango fruits (country of origin: Puerto Rico) were washed under running water, scrubbed with a brush to remove possible pesticide residues, and then peeled. The resulting peels were air-dried for 7 days at room temperature (22–24 °C) and protected from direct sunlight, with a final moisture content of 11.1%. The completely dried peels were ground to a uniform size using a kitchen blender (Blender B1004E, Gorenje, Slovenia) at 1400 W and 32,000 rpm, and the mean particle size was determined by sieve analysis to be 1.0 mm. Ultrasound-assisted extraction (UAE) with pure EtOH was used to extract the bioactive compounds from the dried mango peels, as described in detail in our previous study [24]. The ratio between EtOH and dried mango peels was 10:1. After the extraction procedure, EtOH was evaporated using a rotary evaporator (Büchi^®^ Rotavapor R-144, Flawil, Switzerland). The extract was stored in a freezer at −18 °C until further analysis.

### 2.4. Preparation of MPE-Loaded Alginate Beads

**Figure 1:**
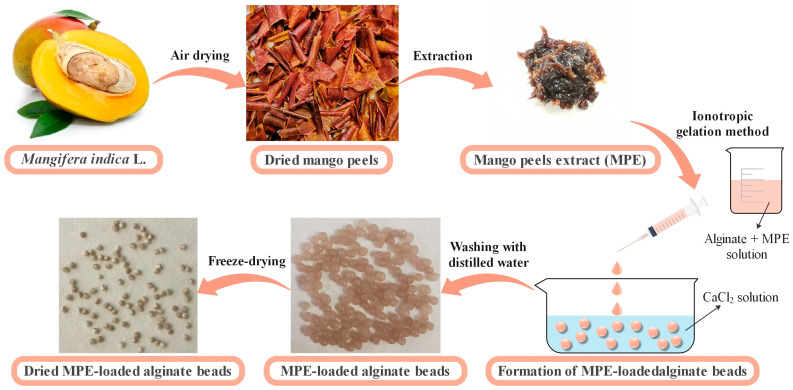
Schematic representation of MPE-loaded alginate bead preparation by ionotropic gelation method.

Encapsulation of MPE in alginate beads was performed to stabilize its important bioactive compounds [1]. The alginate beads were prepared using the ionotropic gelation technique [36]. The beads were formed by adding the suspension of alginate and MPE dropwise into a CaCl_2_ solution (0.1 M) with constant stirring. The control alginate beads without MPE were prepared using the same procedure. The obtained beads were repeatedly washed with distilled water and dried by the freeze-drying process (Figure 1).

### 2.5. Particle Size Determination

The size of alginate beads was determined using the Open Drop algorithm with a Basler Aca1300-200 µm digital camera connected to a computer with a CCTV lens (Tamron, Saitama, Japan). The samples were illuminated and recorded with a camera. The particle size was measured with the ImageJ program (version 1.54d, National Institutes of Health, Bethesda, MD, USA) using 100 beads to determine their diameter (mm). The size distribution graphs were created using the OriginPro^®^ program (version 10.1.0.178, OriginLab Corporation, Northampton, MA, USA).

### 2.6. Encapsulation Efficiency Evaluation

The encapsulation efficiency (EE) of MPE in alginate beads was determined by an alternative method [41] based on the content of non-encapsulated MPE, which was determined by measuring the absorbance using a UV-VIS spectrophotometer (Varian Cary Probe 50, Agilent Technologies, Santa Clara, CA, USA) at a wavelength of 274 nm. The percentage EE was defined as the ratio between the weight of MPE in the beads and the weight of MPE in the initial solution.

### 2.7. Scanning Electron Microscopy (SEM)

SEM analysis was performed using a scanning electron microscope (FE SEM, JSM-IT800, JEOL, Tokyo, Japan) equipped with a secondary electron (SE) detector. The accelerating voltage for imaging was 10 kV. The SEM images were obtained at a magnification range of 40–1000× to examine the morphology of the freeze-dried control and MPE-loaded alginate beads. Before analysis, the alginate beads were coated with a conducting carbon layer.

### 2.8. Thermogravimetric Analysis/Differential Scanning Calorimetry (TGA/DSC)

The alginate beads were subjected to a thermal stability test using TGA/DSC. Both analyses were performed simultaneously on a TGA/DSC instrument (TGA/DSC1, Mettler Toledo AG (MTANA), Zurich, Switzerland) at a 50 mL/min nitrogen flow rate. The samples were weighed in aluminum pans and analyzed at a temperature range of 25–500 °C and a temperature rate of 10 °C/min.

### 2.9. Fourier Transform Infrared Spectroscopy (FTIR)

FTIR analysis was performed to determine the possible chemical interactions within the MPE and alginate beads. The samples were pressed between two ATR diamond crystals. Spectra were acquired in the 4000–400 cm^−1^ range and recorded using the FTIR spectrometer (Shimadzu IRAffinity-1S FTIR spectrometer, Kyoto, Japan).

### 2.10. In Vitro Release Study

The release of the MPE from alginate beads was studied in vitro in simulated gastric fluid (SGF) and simulated intestinal fluid (SIF). SGF and SIF were prepared as in the study of Li et al. [34]. MPE-loaded alginate beads were subjected to SGF (pH 1.2) or SIF (pH 7.4). For in vitro release measurements, alginate beads were incubated at 37 °C under constant shaking at 100 rpm. In the collected sample aliquots, the released amount of the encapsulated MPE was quantified using an analytical method with a UV-VIS spectrophotometer.

The release kinetics of MPE from alginate beads in gastrointestinal fluids were evaluated using various mathematical models using software-supported statistical analysis (OriginPro^®^ (version 10.1.0.178, OriginLab Corporation, Northampton, MA, USA)). These include the zero-order model (which represents the percentage of substance released per unit time), the first-order model (which illustrates the logarithmic relationship between the remaining percentage of substance and time), the Higuchi model (which determines the percentage of substance released as a function of the square root of time), and the Korsmeyer–Peppas model (which determines the logarithmic relationship between the percentage of substance released and time) [45,48].

### 2.11. Determination of Antibacterial Activity

A plate count method was used to determine the inhibitory properties of MPE-loaded alginate beads against *E. coli* and *S. aureus*, representatives of Gram-negative and Gram-positive bacterial species, respectively. Both bacterial cultures in a final inoculum of 10^6^ CFU/mL in nutrient broth were incubated for 24 h at 37 °C in the presence of control alginate beads, MPE-loaded alginate beads, and pure MPE solution. The number of viable bacterial colonies was determined at different time intervals (3, 6, and 24 h) using tenfold serial dilutions and the spread plate technique. The agar plates were incubated at 37 °C for 24 h to determine the bacterial colonies and CFU/mL. The percentage of bacterial reduction was calculated based on the ratio of the difference between the number of colony-forming units per mL of the control and the tested sample and the number of units of the control sample. CIP-loaded alginate beads and pure CIP solution were used as a positive control.

All experiments were performed in three replicates, and the results are expressed as mean values ± SD.

### 2.12. Statistical Analysis

All statistical data analyses were carried out using IBM^®^ SPSS^®^ Statistics software (version 25, IBM Corporation, Armonk, NY, USA). The Shapiro–Wilk test was used to assess the normality of the data distribution. Levene’s test was used to assess the homogeneity of variances. The differences between the groups were determined using a one-way analysis of variance (ANOVA) and a subsequent Tukey’s post hoc test.

## 3. Results and Discussion

Despite many positive effects, sensitive bioactive compounds, including those present in MPE, as presented in our previous study [24], can be easily degraded, which impairs their bioavailability. Degradation can be caused by various factors such as enzymes, pH, heat treatment, oxidation, light, and hydrolysis. To ensure the maximum effect of bioactive compounds, their protection from degradation is a crucial step. At the same time, there is a strong trend towards the use of biocompatible, biodegradable, and non-toxic delivery systems, including biopolymeric particles such as alginate beads, which are particularly suitable for the food industry. Therefore, the stabilization of bioactive compounds from MPE obtained by UAE with EtOH was performed by encapsulation in alginate beads.

### 3.1. Loading Properties of Alginate Beads

Bioactive substances are easily degraded in the gastrointestinal tract and, therefore, cannot be absorbed by the body. However, suitable encapsulation in various carriers, such as alginate beads, can maintain and increase their bioavailability. The EE mainly depends on the affinity between the wall material and the encapsulated substance [49].

In the preparation phase of the alginate beads, they are left in a settling solution for a certain time to solidify before being washed with distilled water to remove the unencapsulated extract from the surface of the beads. The dripping of the alginate solution and extract into the settling solution leads to cross-linking with the formation of a complex coacervate membrane [50]. The most effective gelling agent for the formation of beads is CaCl_2_, as it successfully creates a bond between alginate chains through ionic interactions. The reaction of the carboxyl groups of the adjacent alginate chains with divalent Ca^2+^ ions leads to the formation of a three-dimensional network and, thus, to cross-linking. This leads to the formation of the “egg box” model. In this way, bioactive substances are successfully protected [37].

In a preliminary study, alginate beads were solidified for 5, 15, and 60 min, whereby alginate beads that had been solidified for 5 min appeared to be slightly unstable. On the other hand, there was no significant difference between the 15- and 60-min solidified alginate beads in terms of the EE of MPE (63.1 ± 1.5%–66.0 ± 2.3%) and its in vitro release profile in PBS (pH 7.4, 37 °C), resulting in the release of 88.2 ± 4.2%–98.1 ± 2.9% of MPE after incubation for 24 h in PBS. In addition, the gelation time in the CaCl_2_ solution has a significant influence on the size of the beads but less of an influence on their shape [51]. If the solidification process is prolonged, the resulting beads are smaller, as extensive cross-linking takes place, which increases the rigidity of the bead matrix. To avoid excessive shrinkage and increased polydispersity within the sample, the curing time should be limited to 15–20 min [38].

Therefore, alginate beads with a solidification time of 15 min were prepared for further analysis (EE = 63.1 ± 1.5%). Other studies in which various bioactive substances from natural extracts, such as red dragon fruit peel (EE = 78.6%) [52], plum peel (EE = 62.2%) [46], natal plum (EE = 86.8%) [53], olive leaves (EE = 21.0%) [54], and beetroot leaf and stem (EE = 38.0% and 58.2%) [47], were incorporated also show the good loading properties of alginate beads. Therefore, alginate beads have been proven to be a compatible matrix for the encapsulation of bioactive substances from natural materials, including MPE.

### 3.2. Morphology of Alginate Beads

**Figure 2:**
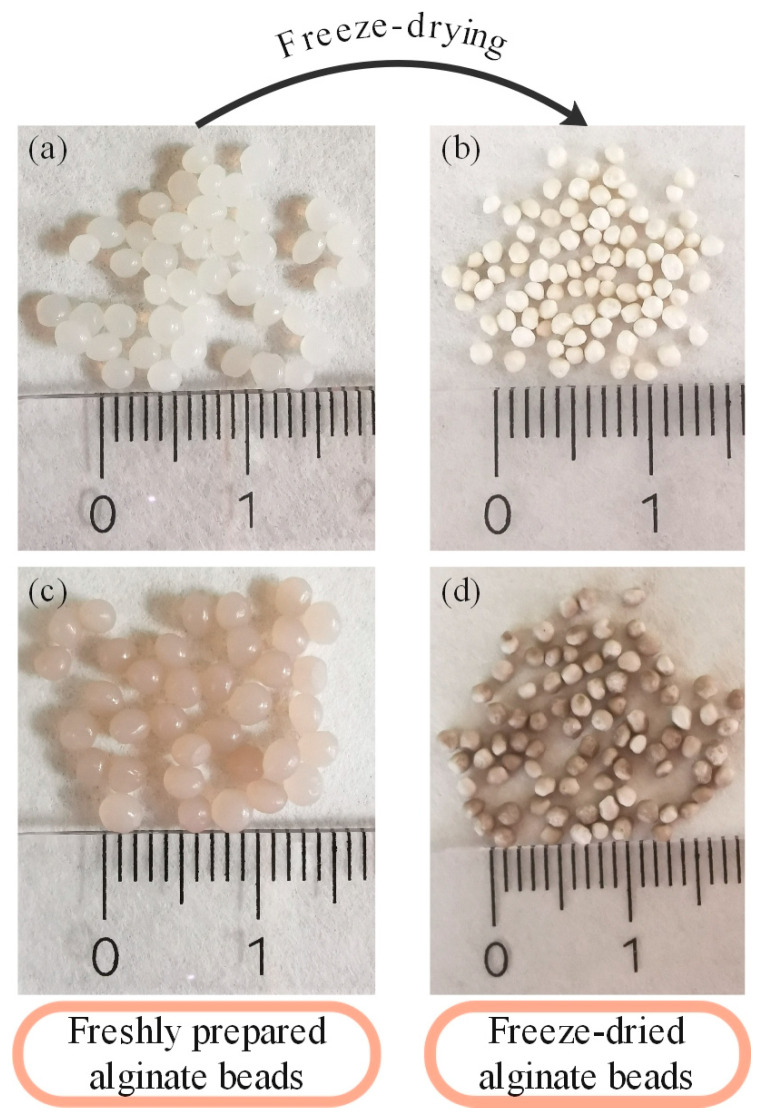
Fresh (**a**) and freeze-dried (**b**) control alginate beads and fresh (**c**) and freeze-dried (**d**) MPE-loaded alginate beads.

The control and MPE-loaded beads produced using the ionotropic gelation method had a spherical or slightly oval shape (Figure 2). 

**Figure 3:**
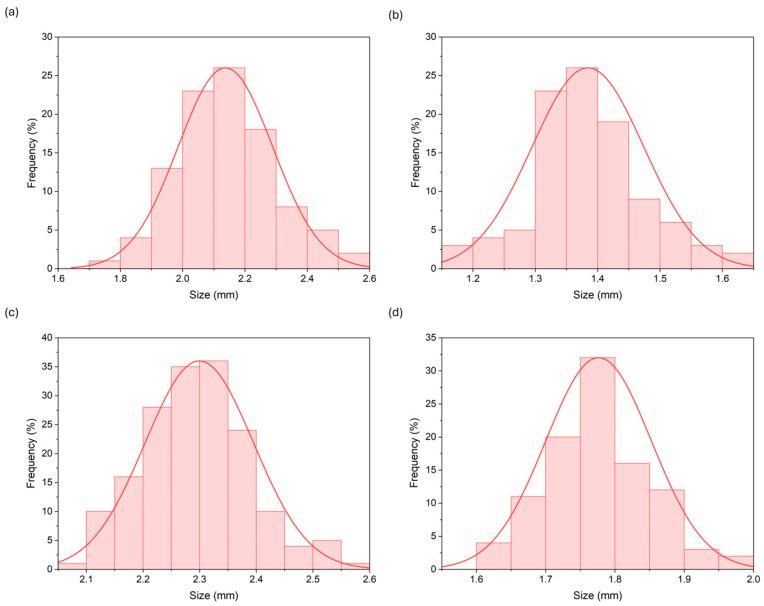
Size distribution graphs of fresh (**a**) and freeze-dried (**b**) control alginate beads and fresh (**c**) and freeze-dried (**d**) MPE-loaded alginate beads. (Size distribution graphs were created using software-supported statistical analysis (ImageJ and Origin Pro).).

The average diameters of the freshly prepared control and MPE-loaded alginate beads were 2.14 ± 0.14 mm (min—max.: 1.70–2.51 mm) and 2.30 ± 0.10 mm (2.09–2.32 mm), respectively. The beads were freeze-dried to extend their shelf life and for further use. After freeze-drying, the average diameter of the control and the MPE-loaded alginate beads changed to 1.38 ± 0.09 mm (1.18–1.61 mm) and 1.78 ± 0.08 mm (1.60–1.78 mm), respectively. The size of the freeze-dried alginate beads was correspondingly smaller compared to the wet ones, which is consistent with other studies [39,41,55]. The frequency of the size distribution for the fresh and freeze-dried control and the MPE-loaded alginate beads is presented in Figure 3.

The overall size of the alginate beads obtained in the present study is in line with the reviewed literature, as the diameters of the beads were larger than 1 mm when using the ionotropic gelation method [41,45,51,56]. Also, the MPE-loaded alginate beads were slightly larger compared to the control beads without MPE. The same increase in bead size was achieved with alginate beads loaded with *Mesona chinensis* extract [36]. Julaeha et al. [45] found that increasing the concentration of the extract leads to a larger diameter of the beads, as the space available for cross-linking with Ca^2+^ ions decreases. In addition, the shape of the alginate beads was insignificantly affected after freeze-drying, as this method successfully preserves the overall roundness of the particles compared to other drying methods [39,41]. However, the surface of beads changed from a smooth to a rough surface, as in other studies [57,58]. The loss of the spherical shape of the beads is due to the breakdown of the water–gel network that occurs during the drying process [59].

The size and shape of beads depend mainly on the needle type and the size of the dripping system; the distance between the needle and the CaCl_2_ solution (settling solution); the gelling conditions; the viscosity, density, and concentration of alginate, CaCl_2,_ and bioactive substances; the flow rate of the polymer solution; and the stabilization time [51,60]. However, the size and shape of the beads are of great importance for the preparation of products in the food industry. For the delivery of encapsulated bioactive compounds, it is desirable that the beads have a spherical geometry with a uniform size distribution [61], a criterion which the beads in our study successfully fulfill.

**Figure 4:**
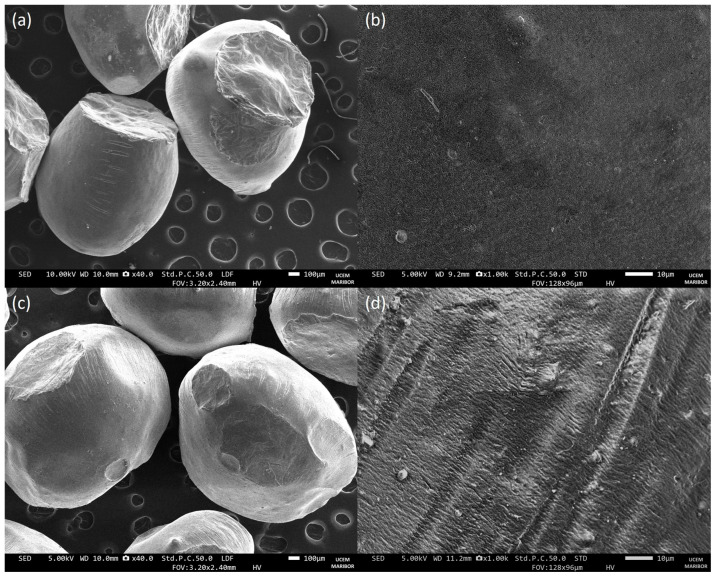
Morphology of freeze-dried control alginate beads at 40× (**a**) and 1000× (**b**) magnification and MPE-loaded alginate beads at 40× (**c**) and 1000× (**d**) magnification under SEM.

Next, the morphology of the freeze-dried control and the MPE-loaded alginate beads was analyzed by SEM (Figure 4). After the freeze-drying process, the surface morphology of the alginate beads was slightly altered. They were still spherical but had an irregular surface. However, there were no cracks on the surface that could impair the proper protection of the encapsulated substances, favor oxygen transport, and thus cause their degradation [62]. In addition, the non-spherical shape of the alginate beads may reduce their strength and, thus, their sensitivity to permeability [36].

The results are consistent with those of other studies, which, however, also report heterogeneous surfaces and the presence of some surface gaps or even cracks [36,41,63]. The surface of the control alginate beads appeared to be smoother than that of the MPE-loaded alginate beads, which had a rougher and wrinkled surface. Furthermore, the size of the alginate beads measured by the ImageJ program was consistent with the diameter of the beads determined by SEM analysis, namely 1.22 ± 0.13 mm and 1.75 ± 0.10 mm for the control and the MPE-loaded alginate beads, respectively.

### 3.3. Thermal Degradation Profile of Alginate Beads

**Figure 5:**
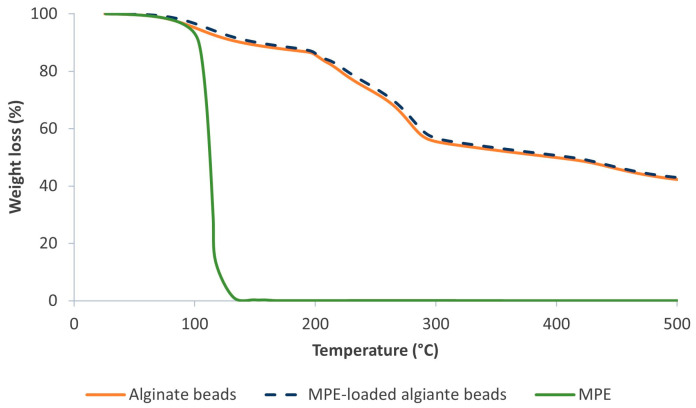
TGA thermogram of control alginate beads, MPE-loaded alginate beads, and pure MPE.

Various extracts and bioactive substances are generally unstable, so their encapsulation is essential. The alginate beads are able to withstand higher temperatures, which improves their thermal stability and effectively controls the loss of the extract [45]. This increases their potential for use in dietary supplements and food processing when exposed to higher temperatures. Therefore, the thermal degradation of the samples (control alginate beads, MPE-loaded alginate beads, and pure MPE) was investigated as weight loss in a temperature range from 20 to 500 °C (Figure 5).

The thermal degradation of polysaccharides involves different stages, namely the desorption of physically absorbed water, the dehydration reaction (the removal of water molecules), and depolymerization, which involves the breaking of C–O and C–C bonds in the ring units leading to the evolution of CO, CO_2_, and H_2_O and the formation of polynuclear aromatic and graphitic carbon structures [39].

The TGA thermogram of pure MPE showed a drastic weight loss in the temperature range from 38 to 157 °C, resulting in a weight loss of 99.6%. On the other hand, four different weight loss phases were observed in the alginate beads (control and MPE-loaded). In addition, when encapsulated in alginate beads, MPE does not degrade too quickly, which is an excellent result in terms of its stability at higher temperatures.

The first weight loss at temperatures of 35 and 150 °C is attributed to the evaporation of water, with a reduction of 11.1% in the control beads and 10.8% in the MPE-loaded alginate beads. During the second phase (190–225 °C) and the third phase (240–315 °C), there was a significant weight loss, namely 36.6% for the control beads and 36.4% for the MPE-loaded alginate beads. This corresponds to the evaporation of the substances, mainly the phenolic compounds in the MPE, as well as the degradation of the polymer [39]. The weight loss during fourth phase at 350–450 °C is due to the decomposition of the polymer network [64]. The residues in the control and the MPE-loaded alginate beads accounted for 42.4% and 42.9% of the total weight of the samples tested, respectively. No significant differences in weight loss were observed between the control and the MPE-loaded alginate beads.

**Figure 6:**
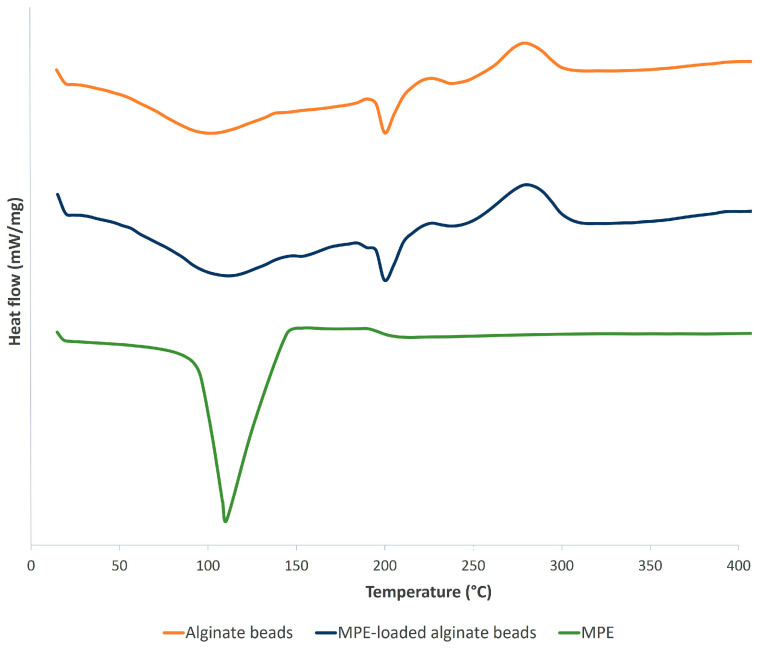
DSC thermogram of control alginate beads, MPE-loaded alginate beads, and pure MPE.

Furthermore, the characterization of the state of alginate beads was examined with DSC analysis, as the synthesis of alginate beads with the ionotropic gelation method involves the transition of the liquid material into a gel [37]. The DSC thermogram of control alginate beads, alginate beads with encapsulated MPE, and pure MPE is shown in Figure 6.

The first endothermic peaks for the control and the alginate beads loaded with MPE were around 104 °C and 112 °C, respectively. The broad endothermic transition temperatures for the control alginate beads and the alginate beads loaded with MPE were 203 °C and 201 °C, respectively, representing the temperature of the transition phase. The endothermic peaks are due to the loss of water associated with the hydrophilic groups of sodium alginate [65].

The exothermic peak of the control alginate beads at around 277 °C and the alginate beads loaded with MPE at around 279 °C resulted from the reactions that take place during the degradation of sodium alginate [66]. Similar peaks were determined for pure sodium alginate powder [31].

A sharp endothermic peak can be seen on the DSC thermogram of the pure MPE due to the melting of the crystalline structure of the MPE at a temperature of 118 °C. The endothermic peak of the MPE-loaded alginate beads has shifted to a higher temperature (201 °C). TGA showed that the encapsulation of MPE in alginate beads can improve the thermal stability of the bioactive substances of MPE against thermal degradation [36]. Similar thermogravimetric profiles were also obtained in other studies where extracts of thyme [39], natal plum [53], and key lime [45] showed improved thermal stability, which helps to reduce the evaporation rate of the encapsulated extract [45].

### 3.4. FTIR Analysis

**Figure 7:**
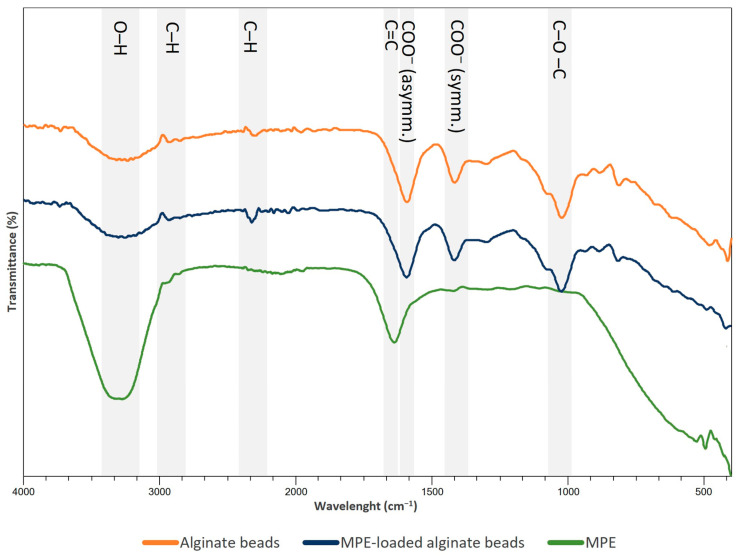
FTIR spectra of control alginate beads, MPE-loaded alginate beads, and pure MPE.

FTIR analysis was conducted to identify possible physiochemical interactions between the alginate matrix and MPE compounds. The obtained FTIR spectra in the range of 4000–400 cm^−1^ of the control, the MPE-loaded alginate beads, and the free MPE are presented in Figure 7.

The FTIR spectrum of the alginate beads showed a characteristic peak at 3250 cm^−1^ corresponding to the O-H stretching band. The weak peak at 2932 cm^−1^ corresponds to the –CH_2_ group, and the peak at 2320 cm^−1^ is due to C–H bending. The peaks at 1640 cm^−1^ and 1438 cm^−1^ indicate strong asymmetric and weaker symmetric vibrations of the carboxyl anion (COO^−^), respectively—both characteristic of alginate carboxyl groups [1]. The peak at 1022 cm^−1^ corresponds to the C–O–C group [39,67]. These characteristic bands present in the control alginate beads are consistent with previous studies [68,69]. The FTIR spectra of the MPE-loaded alginate beads showed a similar profile to the spectra of the control alginate beads, indicating that the MPE was successfully encapsulated in the beads. Representative regions in the MPE-loaded alginate beads include peaks at 3231 cm^−1^, 2920 cm^−1^, 2295 cm^−1^, 1593 cm^−1^, 1416 cm^−1^, and 1022 cm^−1^, corresponding to the functional groups O–H bond, CH_2_ stretching, C–H bending, asymmetric and symmetric COO^−^ stretching, and C–O–C bond, respectively. In addition, no new peak was observed in the MPE-loaded beads. The characteristic peak in the spectrum of pure MPE at 3271 cm^−1^ corresponds to the stretching vibration of the O-H groups, which is characteristic of phenolic compounds [70]. The peaks at 2947 cm^−1^ and 2859 cm^−1^ are due to stretching vibrations of the aliphatic C–H groups, and the peak at 1636 cm^−1^ is due to stretching vibrations of the aromatic C=C groups [68]. The peaks in the 1600–1200 cm^−1^ range are most likely due to the presence of phenolic compounds in MPE [71].

Despite some minor differences in the absorption peaks, it was found that there were no strong interactions between the alginate beads and the MPE loaded into the alginate beads. This indicates that the MPE was encapsulated in the beads without any chemical interactions that could alter the properties of the MPE, and that it is, therefore, relatively stable. These findings are in agreement with other studies, where no chemical interactions were found between alginate beads and plant extracts [1,39,41,45,69].

### 3.5. Release Profile of MPE from Alginate Beads

The encapsulation of bioactive substances can improve their bioavailability during digestion. In this way, they are protected from unfavorable conditions in a specific part of the digestive tract—the stomach—and thus successfully reach the target environment—the intestine—resulting in better absorption by the human body [63]. The release of bioactive substances from biopolymeric carriers is usually influenced by various external factors, such as pH value, ionic strength, solvent and buffer composition, temperature, and pressure [72]. The release of aqueous extracts or hydrophilic substances is generally characterized by a rapid release profile from alginate beads due to their porous structure [73].

**Figure 8:**
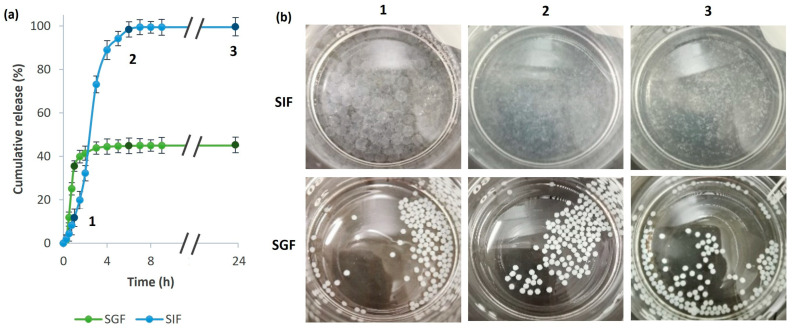
The release profile of MPE from alginate beads in SGF and SIF (**a**) and visual observation of alginate beads during exposure to SGF and SIF at different time intervals (1: 1 h; 2: 6 h; 3: 24 h) (**b**). The different time intervals are indicated in the graph (**a**) by a darker shade of green or blue.

The release profile of MPE from alginate beads was evaluated under simulated gastric conditions (pH 1.2) and simulated intestinal conditions (pH 7.4). The results of the cumulative release of MPE during 24 h exposure to SGF and SIF and visual observation at different time intervals of alginate beads (1: 1 h; 2: 6 h; 3: 24 h) are shown in Figure 8a,b. Although MPE was almost completely released from the alginate beads after 6 h, we extended the release to 24 h to observe the degradation of the polymeric matrix in view of further antibacterial investigations.

The in vitro release study showed an initial steep release of MPE from alginate beads in both simulated gastrointestinal fluids. The initial burst release effect could also be partly due to the un-entrapped MPE adsorbed on the surface of the alginate beads and not completely removed from the surface by repeated washing with distilled water after the formation of the beads.

**Figure 9:**
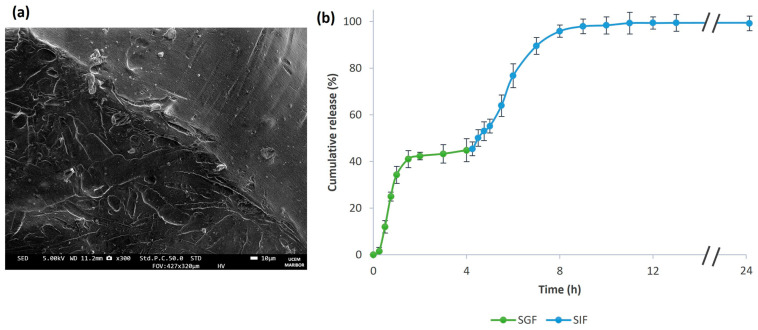
SEM image of MPE-loaded alginate beads after 24 h of exposure to SGF at 300× magnification (**a**) and the release profile of MPE from alginate beads in SGF during the first 4 h and from 4 to 24 h in SIF (**b**).

After 3 h, 43.8% of the MPE was released from the alginate beads during incubation in SGF. Further, the release in SGF began to stabilize. After 24 h, the percentage of MPE released increased by only 1.2%. This indicates that the alginate beads are stable in a very acidic pH range. After the MPE-loaded alginate beads were exposed to SGF, they were freeze-dried and subjected to SEM analysis. The SEM images (Figure 9a) showed tiny cracks on the surface of the alginate beads from which the MPE could be released, suggesting that the initial burst release effect, as mentioned above, could be partly due to the un-entrapped MPE adsorbed on the surface of the alginate beads. However, the tiny cracks visible in the SEM images indicate that the MPE was also partially released from the interior of the alginate beads. However, larger cracks were not present, proving that the beads remained stable in SGF at a lower pH.

On the other hand, the alginate beads began to swell and degrade at higher pH values (SIF) due to the higher solubility of alginate at higher pH values [37], as seen in Figure 8b. As a result, the MPE was successfully fully released from alginate beads in SIF.

The reason why the solubility of alginate depends on the pH value is the presence of carboxyl groups that dissociate or incorporate protons (H^+^). Alginate is negatively charged at a pH above 3.5. It consists of repeating units of (1,4)-linked β-d-mannuronic acid (pKa = 3.38) and α-L-guluronic acid (pKa = 3.65). At a pH below the pKa values of the constituent acids, the alginate polymer incorporates protons, which is the reason for its insolubility [74,75,76]. This was demonstrated when exposed to SGF, where the bioactive compounds of the encapsulated MPE were successfully protected. Above the pKa value, alginate becomes soluble, resulting in the release of bioactive compounds of the encapsulated MPE from alginate beads when exposed to SIF. Therefore, a controlled and sustained release occurred during the incubation of the MPE-loaded alginate beads in SIF. Within 6 h, 98.3% of the encapsulated MPE was released from the alginate beads. After 6 h, no significant difference in the amount of MPE released was observed when the exposure time to SIF was further extended. The release percentage of the MPE from alginate beads after 24 h was 99.4%.

In addition, the MPE-loaded alginate beads were recovered from the SGF after 4 h, as the steady state was reached after 4 h, and then subjected to SIF. It was found that there were no significant differences in the release profile of the bioactive compounds from MPE in SIF (after exposure to SGF) and the release profile directly in SIF. After 24 h of exposure, the release percentage was 99.5%, as shown in Figure 9b.

From the results obtained, it can be concluded that alginate is a good barrier for encapsulated bioactive substances in the acidic pH range and, thus, successfully protects them from being released too quickly. In this way, the bioactive substances reach the intestine, where a sustained release is enabled, and successful absorption into the human body is guaranteed. Other authors reached similar conclusions and reported a similar release profile for different extracts (jabuticaba peel extract [41], *Spirulina* sp. [37], and *Clitoria ternatea* petal flower extracts [69]) from alginate beads in SFG and SIF.

The release of bioactive compounds from a polymer matrix, such as alginate beads, can be controlled by various release mechanisms, including diffusion, swelling, disruption, or a combination thereof [11]. Four mathematical kinetic models were fitted to the experimental data to determine the behavior and mechanism of the release kinetics of the encapsulated MPE from alginate beads in simulated gastrointestinal fluids. The best fit to the experimental data and, thus, the most appropriate kinetic model was determined on the basis of the coefficient of determination (R^2^) [41,45]. Results are presented in Table 1.

**Figure 10:**
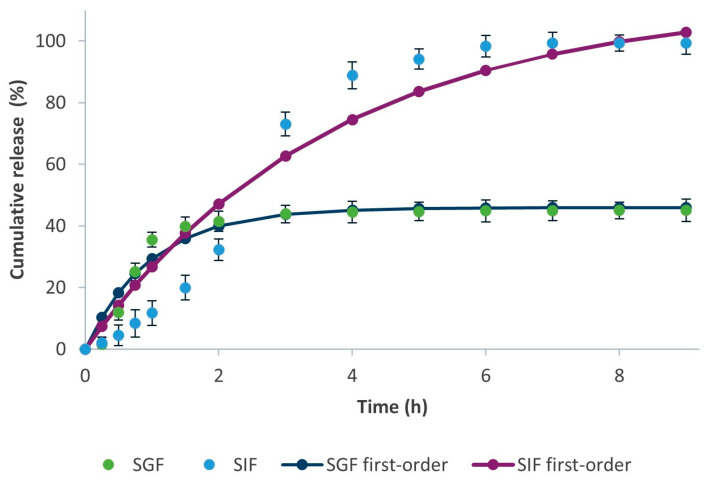
Fitted experimental results to the theoretically predicted release of MPE from alginate beads in SGF and SIF.

The results indicate that the release of MPE from alginate beads in SGF and SIF can be adequately described by the first-order model, as R^2^ was the closest to 1. Figure 10 further presents the experimental results fitted to the theoretically predicted release of MPE from alginate beads in SGF and SIF following the first-order release mechanism, which describes a concentration-dependent release. It shows a typical release of soluble agents encapsulated in porous systems [77].

On the other hand, the Korsmeyer–Peppas model is useful for studying drug release from systems where the release mechanism is unknown or where more than one type of drug release phenomenon is involved. The value of the constant n in the Korsmeyer–Peppas model, the release exponent, predicts the release mechanism of the drug. If the n value is less than or equal to 0.45, the release pattern corresponds to the Fickian diffusion mechanism; if n is between 0.45 and 0.85, it belongs to non-Fickian transport (anomalous transport); if n is equal to 0.89, it corresponds to Case II transport; and if n is above 0.85, it belongs to Super Case II transport for a spherical geometry, such as that of alginate beads [78]. In the case of the release in SGF, the diffusion coefficient n has a value of 0.334, which means that the release mechanism corresponds to Fickian diffusion. Simple Fickian diffusion is a process in which the transport of molecules is driven by a concentration gradient [78].

The release mechanism of MPE in SIF can be as well described as with the first-order model, as the R^2^ was the closest to 1. The n value of 0.870, which fulfills the criterion of n > 0.85, indicates a Super Case II transport that occurs when the solvent diffusion rate is higher than the polymer relaxation [79]. Furthermore, this confirms matrix degradation as a release-controlling factor [80], which is evident from Figure 8b, as the alginate beads began to degrade.

Compared to other studies, a different release behavior of extracts from alginate beads and, consequently, a different agreement with mathematical models was found. The differences in the release behavior of extracts from alginate beads compared to other studies are mainly due to the differences in the extraction methods, the types of extracts and bioactive compounds, the preparation methods of the alginate beads, and the encapsulation method of the extracts [55].

### 3.6. Antibacterial Potential of MPE-Loaded Alginate Beads

Due to the increasing bacterial resistance to various antibacterial agents, there is a growing search for new antibacterial agents, especially those derived from natural materials. As these consist of sensitive bioactive substances, their encapsulation in edible alginate beads, which allows sustained release in the environment at higher pH values, can achieve a successful inhibitory effect against various types of bacteria in a time-released manner.

In our previous study [24], we confirmed the exceptional antibacterial properties of MPE against the growth of *E. coli* and *S. aureus*, which is due to the presence of various phytochemicals and bioactive compounds that disrupt the cell wall of the microbial cells [81]. It is generally considered that Gram-negative bacteria are usually more resistant to antimicrobial agents because they have an outer membrane that Gram-positive bacteria do not possess. Due to the presence of lipopolysaccharides in their membrane, Gram-negative bacteria are more resistant, as they make it more difficult for some antibacterial agents to penetrate. Lipopolysaccharides also repel or slow down interactions with polyphenols [82].

**Figure 11:**
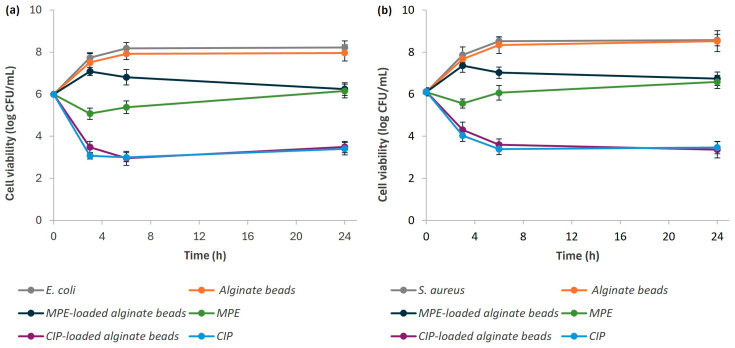
Bacterial growth curves of *E. coli* (**a**) and *S. aureus* (**b**) after exposure to control alginate beads, MPE-loaded alginate beads, free MPE, CIP-loaded alginate beads, and free CIP.

The antibacterial potential of the MPE-loaded alginate beads against *E. coli* and *S. aureus*, representatives of Gram-negative and Gram-positive bacterial species, respectively, was investigated using a plate count method. Both bacterial species are known to be pathogenic, which can also cause intestinal infections [83,84]. A kinetic study was carried out over a period of 24 h at 37 °C. The bacterial strains were exposed to the control alginate beads, the MPE-loaded alginate beads, and the free MPE. CIP-loaded alginate beads and free CIP were used as a positive reference. Figure 11 shows the results of the bacterial growth curves of *E. coli* (a) and *S. aureus* (b) expressed as cell viability (log CFU/mL), and Table 2 summarizes the percentage of bacterial reduction after 24 h of exposure of each bacterial strain to the MPE-loaded alginate beads and free MPE.

The control alginate beads had no significant effect on the growth of the two tested bacterial strains, *E. coli* and *S. aures*, as the bacterial cells were able to grow in a similar way as in the control bacterial suspension. However, it can be seen that the free MPE and the MPE-loaded alginate beads inhibited the growth of both tested intestinal pathogens compared to the control alginate beads and the control bacterial suspension. The same applies to free CIP and CIP-loaded alginate beads used as a positive reference.

Figure 11 and Table 2 show that after three hours of incubation in the presence of free MPE, the number of viable bacteria decreased by 2.7 log for *E. coli* (a 99.8% reduction) and by 2.3 log for *S. aureus* (a 99.5% reduction). After six hours, the tested bacterial species had probably adapted to the bioactive compounds with antibacterial potential in MPE, as a slight increase in bacterial growth was observed. Despite the slight bacterial regrowth, the MPE still strongly inhibited the growth of *E. coli* (a 99.2% reduction) and *S. aureus* (a 99.0% reduction) after 24 h of incubation.

A slightly different trend was observed for the MPE-loaded alginate beads. As a result of successful encapsulation, the inhibition of MPE was gradually achieved as a function of time, which is due to the uniform release of MPE from the beads, a desirable property for carriers of bioactive substances. These results are consistent with the in vitro release profile in SIF (pH = 7.4), as the pH of the bacterial growth media for *E. coli* and *S. aureus* was 6.8 and 7.2, respectively. After three hours of exposure to the MPE-loaded alginate beads, the percentage reduction was only 77.7% for *E. coli* and 69.5% for *S. aureus*. This is consistent with the fact that 73.1% of MPE was released into the SIF in the first three hours, as shown in Figure 8 in Section 3.5. When most of the MPE was released from the alginate beads (98.3%) into the SIF (Figure 8), inhibition of the growth of both bacteria by more than 90% was achieved after 6 h. By prolonging the exposure of both bacterial strains to MPE-loaded beads for 24 h, no significant difference in growth was observed. When encapsulated MPE was fully released from the alginate beads, a similar inhibitory potential was observed when bacterial strains were exposed to MPE in free form, suggesting that antibacterial activities were successfully maintained in the functionalized beads. However, due to the gradual release of MPE from the alginate beads, a gradual increase in bacterial reduction was also observed, while a slight regrowth when exposed to free MPE was observed due to a slight probable adaptation of the bacteria to the compounds from MPE. This indicates that a more favorable inhibitory effect against both bacterial species was observed by encapsulation in alginate beads.

In comparisons, complete inhibition of the growth of both tested bacterial species in the presence of free CIP and CIP-loaded alginate beads was achieved after just three hours.

## 4. Conclusions

Fruit by-products such as mango peels represent a renewable material that could make a significant contribution to the sustainable development of new products with a high content of bioactive substances for biomedical applications, especially in the food industry, to provide antimicrobial activity. Since various bioactive substances are unstable in the gastrointestinal tract, it is crucial for their use in functional foods to incorporate them into edible delivery systems such as alginate beads to increase their shelf life and stability, preserve the bioactive substances, and maintain their bioavailability. In this manner, this study demonstrated that the MPE encapsulated in edible sodium alginate beads could be successfully produced with an EE of 63.1% using the inotropic gelation method.

The alginate beads formed an inert environment in the polymer matrix, as no interactions between the beads and the encapsulated MPE were observed during FTIR analysis. Thermal stability analysis revealed a positive effect of gelation on the physicochemical stability of the encapsulated MPE. Furthermore, the SEM analysis confirmed the absence of cracks on the surface and thus the proper protection of the MPE encapsulated in the beads, possibly ensuring successful preservation even after long-term storage.

Among others, ellagic acid, gallic acid, and catechin are found in the highest concentration in MPE, as determined in our previous study [24]. Since these bioactive compounds have many benefits for human health—they play an important role in the treatment of many diseases, mainly due to their antioxidant, anti-inflammatory, and anti-cancer properties—they play a crucial role in the production of high-value products. Alginate beads serve as a good barrier matrix for encapsulated MPE and its bioactive compounds at acidic stomach conditions due to their stability at lower pH values. Consequently, these bioactive substances can reach the target site, i.e., the intestine, where they are gradually released according to a first-order kinetic mechanism. This leads to the maintenance and improvement of the biological activities, bioaccessibility, and bioavailability of the encapsulated MPE, indicating successful absorption into the human body and, consequently, a better effect on human health, especially in terms of protection against the development of various diseases. In addition, the antibacterial properties of MPE were successfully maintained, with a significant inhibitory effect against the growth of the pathogenic intestinal bacteria Gram-negative *E. coli* and Gram-positive *S. aureus*. Therefore, MPE-loaded alginate beads are a potentially sustainable bioproduct and an efficient oral delivery system that can be used primarily in the food industry, for example, as dietary supplements, functional foods, or food additives. 

In order to question the encapsulation of the extract in alginate beads for large-scale production, the optimization of the processes as a whole is necessary, but at the same time, further investigations are required, especially concerning the cytotoxicity of both the extract itself and the alginate beads as promising edible carrier systems.

## Figures and Tables

**Table 1:** Experimental data fitted to four mathematical kinetic models to determine the behavior and mechanism of the release kinetics of MPE from alginate beads in simulated gastrointestinal fluids (k-the release constant; n-the release exponent; R^2^-coefficient of determination; SE-standard error of estimate).

Model	Zero-Order		First-Order		Higuchi		Korsmeyer-Peppas			Release Mechanism
	R^2^	k_0_	R^2^	k_1_	R^2^	k_H_	R^2^	K_KP_	n	
SGF	0.556	0.091	0.947	0.017	0.732	2.780	0.812	6.855	0.334	Fickian diffusion mechanism
	SE = 0.0256		SE = 0.0027		SE = 0.2098		SE = 2.9633			
SIF	0.924	0.283	0.946	0.002	0.825	4.629	0.932	0.601	0.870	Super Case II transport
	SE = 0.0258		SE = 0.0012		SE = 0.4103		SE = 0.4237			

**Table 2:** Bacterial reduction percentage of MPE-loaded alginate beads, free MPE, CIP-loaded alginate beads, and free CIP. Different letters in the same row indicate significant difference (*p* < 0.05).

Time (h)	Bacterial Reduction Percentage (%)							
	*E. coli*				*S. aureus*			
	MPE-Loaded Alginate Beads	MPE	CIP-Loaded Alginate Beads	CIP	MPE-Loaded Alginate Beads	MPE	CIP-Loaded Alginate Beads	CIP
3	77.7 ± 2.7 ^a^	99.8 ± 2.3 ^b^	100.0 ± 1.2 ^b^	100.0 ± 1.0 ^b^	69.5 ± 3.2 ^a^	99.5 ± 2.8 ^b^	99.9 ± 1.0 ^b^	100.0 ± 0.9 ^b^
6	95.8 ± 3.2 ^a^	99.8 ± 1.8 ^a^	100.0 ± 0.0 ^a^	100.0 ± 0.0 ^a^	96.8 ± 2.4 ^a^	99.6 ± 2.3 ^a^	100.0 ± 0.0 ^a^	100.0 ± 0.0 ^a^
24	99.0 ± 2.0 ^a^	99.2 ± 2.6 ^a^	100.0 ± 0.0 ^a^	100.0 ± 0.0 ^a^	98.5 ± 2.9 ^a^	99.0 ± 2.1 ^a^	100.0 ± 0.0 ^a^	100.0 ± 0.0 ^a^

## Data Availability

The original contributions presented in the study are included in the article, further inquiries can be directed to the corresponding author.
